# Health, Neighborhoods, and School Readiness from the Parent Perspective: A Qualitative Study of Contextual and Socio-Emotional Factors

**DOI:** 10.3390/ijerph18179350

**Published:** 2021-09-04

**Authors:** Lauren E. Futrell Dunaway, Alessandra N. Bazzano, Sarah A.O. Gray, Katherine P. Theall

**Affiliations:** 1 School of Public Health and Tropical Medicine, Tulane University, New Orleans, LA 70112, USA; lfutrell@tulane.edu (L.E.F.D.); abazzano@tulane.edu (A.N.B.); 2 Psychology Department, School of Science and Engineering, Tulane University, New Orleans, LA 70118, USA; sgray4@tulane.edu

**Keywords:** family, neighborhood, built environment, school readiness, community

## Abstract

The objective of this qualitative study was to address existing gaps in the literature by gathering parent perspectives on both health and school readiness in regard to neighborhood context, specifically parents’ perceived level of neighborhood safety and support, on physical health and the behavioral and cognitive domains of school readiness. Focus groups were conducted with a total of 28 parents or caregivers whose children attended Early Head Start/Head Start Centers or who received Special Supplemental Nutrition Program for Women, Infants, and Children (WIC) services in New Orleans, Louisiana during fall 2015. Parents discussed concepts of school readiness, neighborhood, the intersection between the two, and parental stress; however, few expressed a clear connection between their concerns about safety, their own stress, and their child’s readiness for school. Disparities in both health and school readiness exist between both racial and socioeconomic groups in the United States, and this study offers a unique and enhanced understanding of the impact of non-academic factors on the well-being and development of young children.

## 1. Introduction

Early life conditions that are disadvantageous, either socially or economically, have been linked to poorer behavioral and cognitive outcomes in young children, which are also associated with health across the life course [1,2,3,4,5,6,7]. Early poor social–emotional skills and delays in executive functioning have been strongly linked through empirical evidence to mental health (depression), chronic diseases (HTN, CVD, diabetes, obesity), and health behaviors (smoking, drug use) in adulthood [8]. The relationship between early learning and life conditions and health in adulthood is compounded for children of low socioeconomic status due to racial, ethnic, and socioeconomic disparities in child health, neighborhood stress, and child development (i.e., school readiness) [9]. While research on disparities in school readiness specifically has focused on factors such as early care and education environments and quality of the home environment, an additional factor that may be contributing to school readiness disparities is the neighborhood in which a child lives [2,10,11,12,13,14,15,16,17,18,19]. 

There is a substantive body of evidence demonstrating the association between neighborhood and child health and development, even when controlling for family-level characteristics such as income or parent involvement [20,21,22,23,24,25,26,27,28,29,30,31,32,33]. Structural factors related to neighborhood disadvantage such as neighborhood poverty, concentrated disadvantage, exposure to violent crime, or additional neighborhood factors (family structure, average household income, education, employment) have been the primary focus of research in this area [6,21,27,28,30,34,35,36,37,38,39,40,41]. While extensive theoretical literature exists outlining the hypothesized associations between neighborhood and child outcomes, little empirical research exists outlining the specific characteristics and mechanisms that play a role in this association, and even fewer studies have included parent perspectives [7,19,21,30,42,43,44,45,46,47,48,49,50,51,52,53,54]. 

There is a small body of qualitative literature that aims to capture parent perspectives on how the neighborhood they live in impacts their children, but this research has been conducted with either parents of adolescents or parents of children with a wide range of ages, limiting the application of results to early childhood [51,53,54] One study found that the majority of parents of a broad age range of children viewed low collective efficacy as the primary neighborhood factor influencing their children’s behavior, above peer influences, exposure to crime, and quality resources in their neighborhood. Although this study was conducted primarily with parents from upper to middle-class income neighborhoods, the results demonstrated that parents living in low-income neighborhoods were more likely to share perceptions of neighborhood influence. The majority of low-income parents, two-thirds, reported that they believed their neighborhood did affect their child; however, the remaining one-third reported that they did not think that their neighborhood affected their child because their children were too young or that they as a parent provided a buffer [53]. Lastly, qualitative work with parents or caregivers has focused broadly on how neighborhoods impact children, either focusing on general social risk or child outcomes, and not specific components of child development or school readiness [51,53,54]. 

School readiness has only been qualitatively explored in the public health literature in one study with parents of low-income, young children thus far [55]. The main findings from discussions with parents revealed three key themes—how school readiness was affected by their child’s social–emotional health, parent perspectives of how school environments shaped school readiness, and what role the parents play in their child’s school readiness. While this study provided in-depth parent perspectives, and there is the mention that neighborhood was discussed by parents, the study did not identify the impact of neighborhood context as an outcome of specific interest. Newer literature has examined school-readiness through a qualitative metasynthesis approach, although there is limited mention of public health factors, such as neighborhood, and only brief mention of how racism and poverty may impact school readiness [56].

The current study aimed to address existing gaps in the literature by gathering parent perspectives on both health and school readiness in regard to neighborhood context as well as information on how parents’ perceived level of neighborhood safety and support play a role in this relationship. 

## 2. Materials and Methods

Qualitative data were collected from August 2015 to December 2015 with parents of children recruited from two Early Head Start and Head Start facilities and one WIC clinic in New Orleans, LA. Information was collected on parent perceptions of, and buffers and barriers to, school readiness; child health and school readiness; neighborhood and child health and school readiness; and parent’s opinions on how parent stress and parent–child relationships impact health and school readiness. The Consolidated criteria for reporting qualitative research (COREQ) 32-item checklist was used to help ensure transparency and quality throughout [57]. The study was reviewed and approved by the Tulane University Institutional Review Board. 

The methodological orientation and theoretical framework for this study was grounded theory [58,59]. This inductive approach was used to generate a common set of neighborhood factors that caregivers of young children viewed as the as the most important contributors to specific child development outcomes (e.g., physical health, cognitive, and socio-emotional development). Preparation of the focus group guide, data collection design, and analysis was guided by this theory. Analysis began with line-by-line open-coding, identifying in vivo codes, and moving on to axial and selective coding [60]. Key-informant document reviews with selected leaders in the field of early childhood education in New Orleans were used to develop focus group guides and to ensure appropriate context. The qualitative analytic guide used can be found in Appendix A. Constant comparative analysis was used and is described further below. 

### 2.1. Participant Selection 

Four focus groups were conducted with primary caregivers of children 0–5 years of age at 3 separate sites. Groups conducted at early learning centers consisted of 4, 7, and 8 people, respectively, and the group conducted at the WIC clinic included 10 participants due to time restrictions. Each focus group was approximately one hour in length, and the final qualitative analytic guide was used to facilitate discussion with all four groups. The final sample included 28 caregivers in total. The youngest child of the caregiver was used as the index child during focus group discussion. Purposive sampling was used to select focus group participants, [61] utilizing both typical case (selection of typical or average cases, in this case a typical parent of a low-income child who attends an Early Head Start/Head Start Center or receives WIC services) and snowball sampling strategies (participants referred other people, with similar characteristics to their own, that they knew with children 0–5 to participate in the focus group) [60]. Caregivers were able to participate if they were told about the study by an Early Head Start/Head Start or WIC staff or were told about the study by another caregiver (as long as their youngest child attended one of the centers or they received services at the WIC clinic), and they agreed to participate following the informed consent process. 

### 2.2. Methods

Audio recordings of focus groups were transcribed, and transcriptions were stored in Microsoft Word. NVivo qualitative analysis software (QSR International, Doncaster, Australia, Version 10 and 11) was used to manage and analyze the qualitative data. Using grounded theory, constant comparative methods were used, with themes being derived from the data. [60] After the first focus group was completed, the research team began to independently review transcripts, using open coding to identify emerging ideas. Focus group guides for the remaining focus groups were modified to include major themes that were covered but may have been omitted from the guide. After conclusion of the groups, and when transcriptions were complete for all groups, open coding continued to identify major themes. Major themes and field notes were used to develop an initial code book (coding tree), moving from individual meaning units to high-order categories. NVivo was used to code each complete transcript with each transcript being independently coded by two people. Cohen’s Kappa coefficient was used to measure inter-rater reliability, and Kappa values ranged between 0.7 and 1.0 for all codes. Memoing was also used to categorize connections and relationships between codes. 

Research team members who participated in transcription (the author and a graduate assistant) met frequently to discuss coding decisions, adjudicate differences, and establish an expanded codebook with primary code categories and sub-themes within each category. Alterations were made to the codebook when a new theme emerged, and codes were refined and collapsed as needed. Then, major themes were compared across caregiver groups. In the results section below, participant quotations are presented to illustrate major themes, and the clarity of major and minor themes is discussed. 

## 3. Results

Table 1 includes sample characteristics for focus group participants. The final sample was 68% African-American, 96% female (either mothers or grandmothers), and approximately 80% were mothers with the remaining 20% grandmothers and one grandfather. Approximately 60% described their family structure as “single-parent”, and parents reporting having anywhere from 1 to 12 children. Participants were divided between parents with children 0–3 years of age (46%) and 3–5 years of age (56%). The majority of the sample was within 18–50 years of age (80%); however, 20% were over the age of 50 (grandparents). Participants living in Orleans Parish were from at least 16 different neighborhoods, which were identified by providing a map of Orleans Parish and asking participants to circle the neighborhood that they lived in on the map. 

Parents identified several major themes across the four focus group discussions including concepts of school readiness, neighborhood, neighborhood and school readiness, and parental stress. 

### 3.1. School Readiness 

Parents defined characteristics of school readiness in two main categories: (1) knowledge content that they believed they should know before going to kindergarten (e.g., alphabet, shapes, colors, numbers) and (2) level of personal independence in the basic activities of daily life (e.g., how to get dressed, how to tie shoes, being potty-trained). Some parents stated that children should know their name, address, and phone number, and a few stated they should be able to write their name.

Course I want my child to be potty-trained, um I want her to know her alphabets, that way she can learn how to read a little, you know, it will be a little easier for her.

-Mother of 1, African American

Because most children, they get to school, they have the knowledge of like knowing their ABCs. Knowing their numbers, stuff like that.

-Mother of 3, African American

Participants also mentioned social–emotional aspects of school readiness and noted skills around listening, respecting adults, and resolving conflict. Participants from one group mentioned social–emotional skills explicitly, while participants in other groups mentioned social–emotional skills (such as paying attention) more indirectly while discussing other topics.

Listening, following, knowing who to talk to. Cause the biggest thing for me when my daughter started was like, we teach our kids [to be wary of] strangers and things like that. So when they go to school it’s like everybody is a stranger. So you have to teach them, like, this is who you need to go to, this is how you identify this individual and things like that.

-Mother of 2, African American

A minor theme that emerged was health and school readiness. Parents shared health-related factors that they think contributed to school readiness including hunger, sleep, and routine/structure.

If they aren’t physically ready and healthy, they won’t do well, they won’t be prepared to learn.

-Mother of 2, White

Parents expressed concerns about how early children must be at school and how late they go to bed, and how that may affect their ability to learn. One of the groups discussed foods that were served at their child’s center and how they felt the children were hungry when they came home, affecting their ability to learn. Parents agreed that structure and routine are beneficial to children.

You got to get up at the same time. You go to bed every night. I think a routine helps young children especially. So, nothing is a surprise.

-Grandfather of 1, White

### 3.2. Neighborhood

When asked how they perceive the concept of their neighborhood, parents across all four groups responded in one of three ways: (1) referencing a geographical area, (2) describing crime, and (3) describing people who lived in the neighborhood. In the one group that did not take place in Orleans Parish, most respondents stated that they thought of their neighborhood as the street or geographical area where they lived. In the three groups that took place in Orleans Parish, many immediately responded with the word “crime” describing how unsafe they felt in the neighborhoods where they lived.

When I think about my neighborhood, I’m kind of like with ******. I think about the crime rate. Um, I stay around the corner from a park. I don’t bring my kids to it cause it’s a wide-open park. There’s a grocery store on the opposite side where they’re like people always hanging out. Somebody’s getting shot. They’re having fights. I don’t have the time to take off running with me and my kid.

-Mother of 2, African American

Within this perception of neighborhood as closely related to the crime, neighborhood safety was discussed in all four groups in great detail. Parents discussed how they feel unsafe in their neighborhoods and how that determines whether they let their children go outside, where they let them play, and what they are able to do with them in their neighborhoods.

Because we be locked up in the house. I’m too scared to be peeking outside my door and a bullet cross my head or something. Me personally, I don’t let my baby go outside because they got too much crime going on and I don’t want nothing to happen to them. So, if he go outside, we just right there at the house. We’re not walking to the park, no not like that. I don’t trust it.

-Mother of 6, African American

Others, when asked to describe their neighborhood, began to talk about people in their neighborhoods and to describe their relationships with them or how they felt these individuals had an influence on their children. Some described keeping to themselves, and others spoke of positive and negative influences.

My neighbors don’t come outside, like we only see them going or coming.

-Mother of 6, African American

Like I said earlier, with you know the different neighbors, you know, [how they have] a positive impact [on my children], asking them, you know, how they [ask them how their] day was or what they learned, so that’s something that’s refreshing they memory.

-Mother of 2, African American

Older participants also talked about how things used to be and how they had changed, regarding both crime and the ability to trust people in the neighborhood.

Nowadays it’s so hard to trust people with your children

-Grandmother of 5, African American

A minor theme that emerged within perception of neighborhood and its influence on children was neighborhood disorder. Participants discussed how some residents had a “disrespect” for their neighborhoods because of litter, broken glass, etc., and commented on how that affected their children in terms of both safety and physical activity. Both structural disorder (litter, blighted buildings and lots) and social disorder (people littering, selling drugs, loitering) were discussed.

Because if you come outside and you got to be looking trifling or anything or they just throwing anything on the ground or they just doing everything in the neighborhood. It’s the people that live in it. Those people neighborhood they making it look worser than what it is.

-Mother of 3, African American

Well, it limits their ability to go outside and play, I mean, you can’t let them out of your sight, you can’t hardly go outside and not step on broken glass, cans, trash, and I mean, people take no respect to their neighborhoods. I’ve seen a garbage can right there and somebody just take their McDonald’s stuff and threw it on the ground.

-Grandfather of 1, White

### 3.3. Neighborhood and School Readiness 

When asked if they thought that the characteristics of neighborhoods that were described (e.g., crime, people who lived in the neighborhood) had an impact on how ready their children were for school, many participants discussed that they thought it was the people living in the neighborhoods, rather than the physical characteristics of the environments that influenced their children.

It’s not where your kids grow up at, it’s what they parents teach them and what’s going on. Cause everybody, everybody said like, oh places they stay at is bad. It’s not the area that’s bad. It’s the people that reside in those areas that make those areas look worser than what they are.

-Mother of 3, African American

Parents voiced that no matter where or what neighborhood that their child was from, that there was still the potential for them to be school ready, and frequently heard in all four groups was the phrase “it starts at home.” When specifying activities that they participated at home in order to prepare their children, parents referred to academically-oriented activities that they did with their children such as flash cards, worksheets, and tablet games and consistently mentioned academic school readiness skills (ABCs, 123s, colors, shapes). Beyond academic school readiness preparation, participants talked about how they chose to discipline, teach, and socialize their children, and how that factor was the most important thing in terms of child-wellbeing and school readiness. Parents expressed hope that regardless of the quality of neighborhood that the child is from/was raised, in that neighborhood can be overcome by the influence of the parent–child relationship and activities with the child.

It’s more about how you raise your child.

-Mother of 3, African American

It all goes back to starting at home, everything with your child, whether it’s now or whether they 50 years old in life. But at the end of the day, every step they take, everything they learning and producing and developing is coming out of your household.

-Mother of 2, African American

While parents most often replied “no” to the direct question “do you think your neighborhood impacts your child being ready for school?” or “do you think your neighborhood is connected to how ready your child is for school?”, parents nonetheless described intentional efforts that they took to protect their children from perceived potential for the neighborhood to have negative impacts on children. Their responses focused on how neighborhood impact could be altered, or made less important, by academic work and socializing practices at home with the child. 

For example, a common theme that was consistent in all four groups in terms of neighborhood impact on children was child safety.

I think going back to how it might affect children in school, I think it creates distractions, worries for them that they shouldn’t really have to have at that age. You shouldn’t have a child of three, four five, any age really, worried about who’s got a gun. They shouldn’t have to worry about dangerous trash on the street. So it’s a distraction. They get to school and their mind is not there in school.

-Mother of 6, African American

Thus, parents described ways in which they perceived that neighborhood safety and context might impact children’s focus on and preparedness for school, as well as intentional home-based approaches to “raising” children that they take to counteract these potential impacts.

### 3.4. Parent Stress 

Child safety was often mentioned in relation to parent stress. Parents spoke of concerns about their child’s safety in relationship to their neighborhood and of their personal responsibility to keep their children safe.

Because she can’t go out and play, I don’t allow my child to go outside, you know and enjoy being a child because of what’s going on in the neighborhood. And I’m afraid, you know, even if we are in the house, bullets don’t have a name on it or it flies anywhere. It’s just if she’s right there.

-Mother of 1, African American

No, I don’t like them to be over there, for them to be outside around there, no because they got a boy that got killed in broad daylight. Right there on the corner where his daddy hang at and that was one of his daddy friends. And I told him, if my kids come over here, keep them inside. I said you don’t have no reason to be outside when all that stuff going on.

-Mother of 3, African American

When asked if they felt like they served as a buffer between their neighborhoods and their children, parents reflected on their perceived responsibility to their children and the stress that they take on to protect their children from situations and people in their neighborhoods.

I don’t serve as a buffer; I manipulate the environment to fit ****’s needs for what

I want him to see. We never use the ****** bus stop. Not one day not ever not never. It is so rare. Because they are all selling drugs, and drinking, and partying and smoking right there at the bus stop. They gone pull out a big bag of weed and I was like alright let’s go!

-Mother of 1, Mixed Race

I’m superwoman. I am super mama. I don’t play. And my children know, with all this foolishness. That’s why I’m asking, what are you seeing, what are you watching, you have to be like the military person now. You don’t want to just be mama no more. You have to be police mama.

-Mother of 6, African American

Parents described feelings of loneliness when it came to raising their child and spoke of needing “a village” to raise their child, but not feeling like they could trust people to help.

We need the village base. It takes a village to raise a child. But you have to filter through five hundred bad to get one good, but that’s how the world is.

-Mother of 2, African American

It’s so hard to trust other people with your kids, you know to not know that. If it’s a group of us, like us four, and we could stay in like one little residential neighborhood where we all neighbors. So Monday I have the kids, Tuesday **** has them, Wednesday you have them. Like it’s a good idea because the community does need to come together, you know, to kind of help each other out along the way to provide support.

-Mother of 2, African American

Parents in all four groups discussed themes of child safety and parent stress in terms of neighborhood, but as mentioned above, few expressed a clear connection between their concerns about safety, their own stress, and their children’s readiness for formal schooling. 

## 4. Discussion

This unique study focused specifically on neighborhood and school readiness, rather than neighborhood and general social risk or child outcomes, as previous qualitative studies have done, exploring these links specifically in a low-income population of parents of young children [51,53,54]. Additionally, it provides in-depth parental perspectives on how parents perceive their neighborhood impacts their young children’s readiness for formal schooling, which is a factor that has not been explored in depth in previous research [55]. In discussions with parents and caregivers, we identified four primary themes: school readiness, neighborhood concept (including safety), neighborhood and school readiness, and parental stress in relation to neighborhood impact on children.

Although research examining more distal, non-academic factors, i.e., neighborhood and child health, and their relationship to child development and school readiness has increased, the construct of school readiness has not always been clearly and consistently defined [34,62,63,64,65]. Recent research approaches to conceptualizing readiness have been more holistic and include components such as physical well-being, social–emotional development, and child behavior; however, the more traditional and most commonly used definitions of school readiness are academic and cognitive in nature [34,41,62,64,65,66,67,68,69]. Consistent with this traditional definition, many parents in this sample described school readiness in terms of rote memory skills rather than relational or social–emotional skills. This conceptualization of readiness could explain why participants rarely articulated specifically how neighborhood impacts school readiness and instead focused on home-based, academic contributions to children’s readiness. More contemporary quantitative research has explored distal factors, including children’s neighborhood structural and social environments, linking them to children’s developmental outcomes, including school readiness and social–emotional development [20,21,22,23,24,25,26,27,28,29,30,31,32,33,51]. However, it may be more difficult for parents to see a connection between neighborhood and the rote memory skills they define as central to school readiness rather than between their neighborhood and other, softer components of school readiness that are documented in quantitative research, such as social–emotional development. 

Parents did not view the physical aspects of the environment, but more often the people living in the neighborhood, as the mechanism through which their neighborhood could influence their child. However, for the most part, parents were hopeful and viewed neighborhood concerns as something that could be overcome by parental factors such as their own relationship with their child or activities they did with them at home. Individual and household-level interactions were perceived as paramount for their children’s well-being. The commonly heard phrase “it starts at home” in this study population was aligned with pre-academic and rote memory skills as a measure of school readiness, which was described as something tangible that parents worked on with their children (games, flashcards, etc.). 

In contrast to Galster and Santiago’s study, which documented parents’ perceptions of neighborhood on older children’s development, ours was derived solely from parents of children 0–5 years of age [53]. Nonetheless, parents in this study similarly discussed crime and the influence of neighbors on their children. Dissimilarly, in our study, crime and neighborhood safety were most commonly mentioned (compared to collective efficacy) as the main neighborhood-level factors influencing their children. Parents discussed how feeling unsafe in their neighborhoods affected their parenting behavior, including whether they let their children go outside, where they let them play, and what children and parents can do in their neighborhoods. This influence of neighborhood crime is also well documented in quantitative studies that have demonstrated that perception of safety influences not only parenting practices but also positive school outcomes for children [70,71,72] Child safety was commonly viewed as at risk, and parents perceived it as their individual responsibility to keep their child safe, in contrast to collective efficacy perspectives found in previous qualitative work or reliance on public infrastructure to make neighborhoods safer [53]. 

While parents did not explicitly verbalize the connection between neighborhood and school readiness, parents often discussed their perception of neighborhood (crime or the people in their neighborhood) in terms of how it was connected to their own stress. The tensions between parental responsibility for child safety, neighborhood structure, lack of perceived support from neighbors, resources, and safety were apparent stressors as parents discussed their neighborhoods and their children. This was compounded by discussion of systemic issues and how parents must be ever more vigilant not only because of the structural challenges but also systemic issues such as lack of police intervention or activities for children in their neighborhoods. How perception of neighborhood and neighborhood structure impacts parenting has been documented in the literature; however, the juxtaposition of agency and structure, specifically for parents of young children, should be examined in greater detail [73]. Although parental responses differed in regard to if they felt like they served as a buffer between their children or not, each parent described their own stress related to limiting exposure to neighborhood crime or protecting their children. These findings resonate with hypotheses that neighborhood factors may act through parent stress to impact children, though again, empirical quantitative evidence is limited for this hypothesis [30,37,74,75,76]. 

The discrepancies between parental perceptions and existing literature on several of the themes warrant further exploration, including definition of school readiness and the connection between neighborhood impact on school readiness. Most of the existing published quantitative research, and unpublished research from a similar cohort, explore the impact of neighborhood social support and how it may operate within families to impact school readiness, which is operationalized as child behavior and social emotional development [21,27,29,77]. In this study, parents viewed pre-academic and rote memory skills (ABCs, 123s) as the key components of school readiness. Furthermore, even though the relationship between neighborhood and child development is well researched, few studies have included parental perspectives, and as seen in this study, parents reported differing definitions of school readiness and a limited perceived impact of neighborhood on their children’s academic abilities [20,21,22,23,24,25,26,27,28,29,30,31,32,33,51,53,54]. It is imperative to have clear definitions and parental perspectives to fully understand the specific characteristics and mechanisms that play a role in the association between school readiness and neighborhood.

Limitations of this study include restriction to a sample of parents in New Orleans, Louisiana, and a subsample of races/ethnicities represented in the larger population served by the organizations through which participants were recruited, presenting a less exhaustive understanding of the potential impact of neighborhood on health and school readiness from different cultural perspectives. Additionally, the lead facilitator of the focus groups did not have direct experience of their neighborhoods, which may have limited their ability to interpret meaning associated with that concept. Limited resources also restricted the number of focus group discussions, and additional data collection may have allowed for a deeper exploration of themes. Lastly, the term “school readiness” itself is an abstract concept with diffuse definitions across the research literature, and parents’ own definitions may not reflect the construct as represented in the broader literature.

## 5. Conclusions

This study not only highlighted parent perspectives on neighborhood factors and facets that influence school readiness but also reinforced the need to include parent perspectives when developing early childhood health and education interventions. Developing a consensus between researchers’ and parents’ definitions of school readiness (child behavior/social–emotional development vs. pre-academic and rote memory skills) is needed to elevate the concept as a key child development outcome that has the potential to impact health in adulthood and both the public health and education fields through its connection to education and employment. Practitioners and researchers should take parent perspectives into consideration, as these may shed light on the mechanisms between non-traditional contributors to disparities in school readiness such as neighborhood. 

## Figures and Tables

**Table 1:** Focus group participant characteristics.

Characteristic	Parents (*N* = 28)	%
**Age**		
18–23	1	0.04
24–29	8	28.57
30–34	6	21.43
34–39	6	21.43
40–50	1	0.04
50+	6	21.43
**Race**		
Asian	2	7.14
Black/African-American	19	67.86
White	4	14.29
Other	3	10.71
**Education**		
Less than High School	3	10.71
High School Diploma or GED	10	35.71
Some College but no Degree	6	21.43
Associates Degree	6	21.43
Bachelor’s Degree or Higher	2	7.14
**Employment**		
Employed Full Time	6	21.43
Part Time	10	35.71
Unemployed/Not in Labor Force	8	28.57

## Appendix A

**Table A1:** Quantitative Analytic Guide.

Specific Aim	Focus Group Topics	Focus Group Question Re-Writes	Analytic Questions
To determine the impact of neighborhood context, specifically parents’ perceived level of neighborhood support, on physical health and behavioral and cognitive domains of school readiness. Hypothesis: Parents of young children will identify a common set of neighborhood factors as the most important contributors to physical health as they relate to school readiness.	Parent perceptions of school readiness	When did you start to think about where your child would go to Kindergarten? What are some of the things that you think are important for your child to know/be able to do before they go to Kindergarten? What do you think it means for your children to be “ready” for school (Kindergarten)? Probe/clarification-How would you explain what it means to be ready for school to another parent?	Q1. Do parents understand the importance of early childhood (experiences, exposures, and environment) to school readiness?
	Child health and school readiness	How do you think your child’s health is connected to how ready they are for school? * Probe-physical health = physical conditions but also could probe on nutrition (food insecurity) and other health behaviors (physical activity, others?) and environmental factors in the home (smoke exposure, violence exposure, environmental exposures (lead, air quality)	Q2. Do parents identify a connection between child health and school readiness?
	Neighborhood and child health/school readiness	What do you think of when I say “your neighborhood?” (do they think of their street, a geographic area, or the people?) What is it like to live in your neighborhood? * * Probe: Do you feel safe in your neighborhood? Think about the people in your neighborhood. Tell me how you feel about how they do or do not support your family and your child. * *Probe: Do you feel like you can count on them? Do you feel like you can trust them? Do you feel like people in the neighborhood might be a bad influence on your child? Can you tell me about some things in your neighborhood that you think might impact your child’s health? * * Probe: people in the neighborhood, quality of the neighborhood, crime, physical spaces, access to healthy food Can you tell me about things in your neighborhood that you think impact your child being ready for school? * * Probe: people in the neighborhood, quality of the neighborhood, crime, physical spaces, access to healthy food When you think of your child’s childcare/preschool, how do you think that impacts how ready your child is for school? What do you think has more of an impact on school readiness—childcare/preschool or home/neighborhood? Why?	Q3.: What common set of neighborhood contextual factors do parents identify as the most important contributors to physical health and well-being as it relates to school readiness?
	Parent stress → health and school readiness	What are some things in your life that you think make it harder for you to help your child be healthy? * Probe-stressors at the individual, family, neighborhood, community levels What are some things in your life that you think support (or help) you to help your child be healthy? What are some things in your life that you think make it harder for you to help your child be ready for school? What are some things in your life that you think support (or help) you to help your child be ready for school? * Probe-relationship with your child, activities with your child	Q4. What do parents perceive as the primary buffers/barriers that contribute to school readiness? What role does parent stress play in child health/development/readiness? OR Does the parent child relationship act as a protective factor that fosters health/development/readiness? Are these separate?
